# The Amagugu Intervention: A Conceptual Framework for Increasing HIV Disclosure and Parent-Led Communication about Health among HIV-Infected Parents with HIV-Uninfected Primary School-Aged Children

**DOI:** 10.3389/fpubh.2016.00183

**Published:** 2016-08-31

**Authors:** Tamsen J. Rochat, Joanie Mitchell, Alan Stein, Ntombizodumo Brilliant Mkwanazi, Ruth M. Bland

**Affiliations:** ^1^Human and Social Development, Human Sciences Research Council, Durban, South Africa; ^2^Africa Centre for Population Health, University of KwaZulu-Natal, Somkhele, South Africa; ^3^MRC/Developmental Pathways to Health Research Unit, School of Clinical Medicine, Faculty of Health Sciences, University of Witwatersrand, Johannesburg, South Africa; ^4^Section of Child and Adolescent Psychiatry, Department of Psychiatry, University of Oxford, Oxford, UK; ^5^MRC/Wits Rural Public Health and Health Transitions Research Unit (Agincourt), School of Public Health, Faculty of Health Sciences, University of Witwatersrand, Johannesburg, South Africa; ^6^School of Public Health, Faculty of Health Sciences, University of Witwatersrand, Johannesburg, South Africa; ^7^DST-NRF Centre of Excellence in Human Development, University of Witwatersrand, Johannesburg, South Africa; ^8^Institute of Health and Wellbeing and Royal Hospital for Sick Children, University of Glasgow, Glasgow, UK

**Keywords:** HIV disclosure, South Africa, children, intervention, conceptual framework

## Abstract

Advances in access to HIV prevention and treatment have reduced vertical transmission of HIV, with most children born to HIV-infected parents being HIV-uninfected themselves. A major challenge that HIV-infected parents face is disclosure of their HIV status to their predominantly HIV-uninfected children. Their children enter middle childhood and early adolescence facing many challenges associated with parental illness and hospitalization, often exacerbated by stigma and a lack of access to health education and support. Increasingly, evidence suggests that primary school-aged children have the developmental capacity to grasp concepts of health and illness, including HIV, and that in the absence of parent-led communication and education about these issues, HIV-exposed children may be at increased risk of psychological and social problems. The Amagugu intervention is a six-session home-based intervention, delivered by lay counselors, which aims to increase parenting capacity to disclose their HIV status and offer health education to their primary school-aged children. The intervention includes information and activities on disclosure, health care engagement, and custody planning. An uncontrolled pre–post-evaluation study with 281 families showed that the intervention was feasible, acceptable, and effective in increasing maternal disclosure. The aim of this paper is to describe the conceptual model of the Amagugu intervention, as developed post-evaluation, showing the proposed pathways of risk that Amagugu aims to disrupt through its intervention targets, mechanisms, and activities; and to present a summary of results from the large-scale evaluation study of Amagugu to demonstrate the acceptability and feasibility of the intervention model. This relatively low-intensity home-based intervention led to: increased HIV disclosure to children, improvements in mental health for mother and child, and improved health care engagement and custody planning for the child. The intervention model demonstrates the potential for disclosure interventions to include pre-adolescent HIV education and prevention for primary school-aged children.

## Introduction

Children living in Southern Africa are rarely left unaffected by the HIV epidemic (1). Recent estimates (2003–2011) using Demographic and Health Surveys from 23 countries in Africa found the largest numbers of children (ranging from 14 to 36%) living with an HIV-infected adult were in Southern African countries (2). This HIV-infected adult was commonly one of their parents, most frequently their mother. Given the high prevalence of HIV in South Africa, the exposure to parental, familial, and household HIV is likely to be much higher than other countries in Southern Africa (3), with a greater number of children in the care of an HIV-infected parent.

Significant improvements in access to antiretroviral therapy (ART) in women of child-bearing age have substantially reduced vertical HIV transmission to children (4, 5). However, a growing body of evidence suggests that HIV-exposed children face a range of risk factors (parental ill health, hospitalization, and ultimately possible death and loss), which impact negatively on their psychological well-being (6, 7). This presents longer-term challenges for the ongoing care and support of HIV-infected parents and their largely HIV-uninfected children over the family lifespan (8–10). A major challenge this growing population of parents will face is when and how to disclose their HIV status to their predominantly HIV-uninfected children (11–14). However, an important opportunity exists to provide and empower HIV-infected parents with the skills and capacity to educate their children about health (including HIV); to teach their children about HIV prevention and the health services available to them; and to actively plan for their child’s future, all of which have been shown to improve children’s outcomes in the literature outside the context of HIV.

Research to date has focused on maternal, rather than paternal HIV disclosure, finding that disclosure of HIV status has been found to be beneficial for mothers, children, and families (12). Much research has focused on rates of disclosure, with a recent systematic review (13) on maternal HIV disclosure reporting rates varying from as low as 10% in some studies to 82% in others, with most finding disclosure rates between 30 and 45%. Although the largest population of HIV-infected parents live in Africa, only a handful of disclosure studies focused on parent disclosure to HIV-uninfected children have taken place in Africa (13, 15, 16). In comparison, international literature shows a rapidly growing body of evidence, including emerging work from China (17–19). Despite this, the literature remains limited overall, with most studies being descriptive and in resource-rich settings, with few interventions in low- and middle-income countries (LMICs) (16) and with most focused on HIV disclosure, without much attention to the broader health education and prevention needs of children.

Interventions facilitating disclosure of either maternal or paternal HIV status to children have a broader potential to mitigate risk factors facing HIV-exposed children. Support for this can be drawn from conceptual models of familial resilience (20), which provide insight into resilient adaptation (21, 22) for children and families, emphasizing the importance of families’ abilities to make meaning of a difficult situation (23) and to create a coherent narrative for children (24). While many parents may be willing to disclose their HIV status, they may not feel confident about how to clearly construct this narrative in a developmentally sensitive manner (8, 25). Thus, a key gap in the literature in sub-Saharan Africa is how parental HIV disclosure should be undertaken and how best to help HIV-infected parents to do this.

Most existing interventions, developed in high-income countries (HIC), take disclosure as an endpoint, and assume that the benefits of this are predominantly in the realm of improving parent and child mental health. In HIV-endemic communities, disclosure may better be conceptualized as a starting point, rather than an end point, with potential to use the disclosure to facilitate parenting capacity to educate children on HIV prevention or to initiate parent-led sex education or custody planning, both of which are known to improve the child’s immediate outcomes and later adolescent outcomes in LMICs. The Amagugu intervention has this aim; it is a family-centered disclosure intervention providing support to HIV-infected mothers to disclose their status to their HIV-uninfected primary school-aged children and to educate them about health and HIV. This paper has two aims: (i) to describe the proposed pathways of risk that Amagugu aims to disrupt through its intervention targets, mechanisms, and activities and (ii) to present a summary of results from the large-scale evaluation study of Amagugu (25, 26).

## Materials and Methods

In the early stages of the development of this intervention, we used the UK Medical Research Council guidelines for developing complex interventions (27) and undertook phased research work to fully develop and test our intervention model. The design was informed by an extensive review of existing evidence and this was followed by piloting and refinement of the intervention with community consultations.

Our review on maternal HIV disclosure to HIV-uninfected children is published elsewhere (28) and summarizes 58 studies, including two literature reviews (11, 12) and a recent systematic review (13). In addition, we reviewed the recent guidelines from the World Health Organization on HIV disclosure to children (29), which included the available evidence on maternal disclosure to HIV-uninfected children of primary school-age, and highlighted the lack of studies in this area. Following this review of the evidence, and given the lack of intervention models available for adaptation, we undertook the development of a clear conceptual framework that would guide intervention design. First, we identified the risk pathways outlined in the literature and formative work; second, we identified potential modifiable intervention targets to establish an intervention pathway that could disrupt these risks. Finally, we designed sessional content that we hypothesized would result in the changes sought through maternal HIV disclosure.

Importantly, the conceptual framework has been informed not only by what we know about how HIV impacts on parenting behavior and child outcomes but also our understanding of parenting capacities and stressors in the context of other parental terminal illnesses. The development of the model has been influenced by family resilience literature and the socio-cultural context within which the intervention was to be tested and delivered. Our intervention targets HIV-infected mothers (as opposed to fathers or other caregivers) for pragmatic reasons, as the vast majority of children are resident with, and cared for, by their biological mothers in our context (30). The intervention is, however, highly adaptable to use with fathers and other caregivers, as outlined in the section on the intervention principles.

## Results

Results are presented in two sections: the first includes the design of the intervention, outlining the conceptual model, intervention targets, and principles; and the second, a summary of the results of the evaluation study.

### Design of the Intervention

#### Conceptual Model

**Figure 1:**
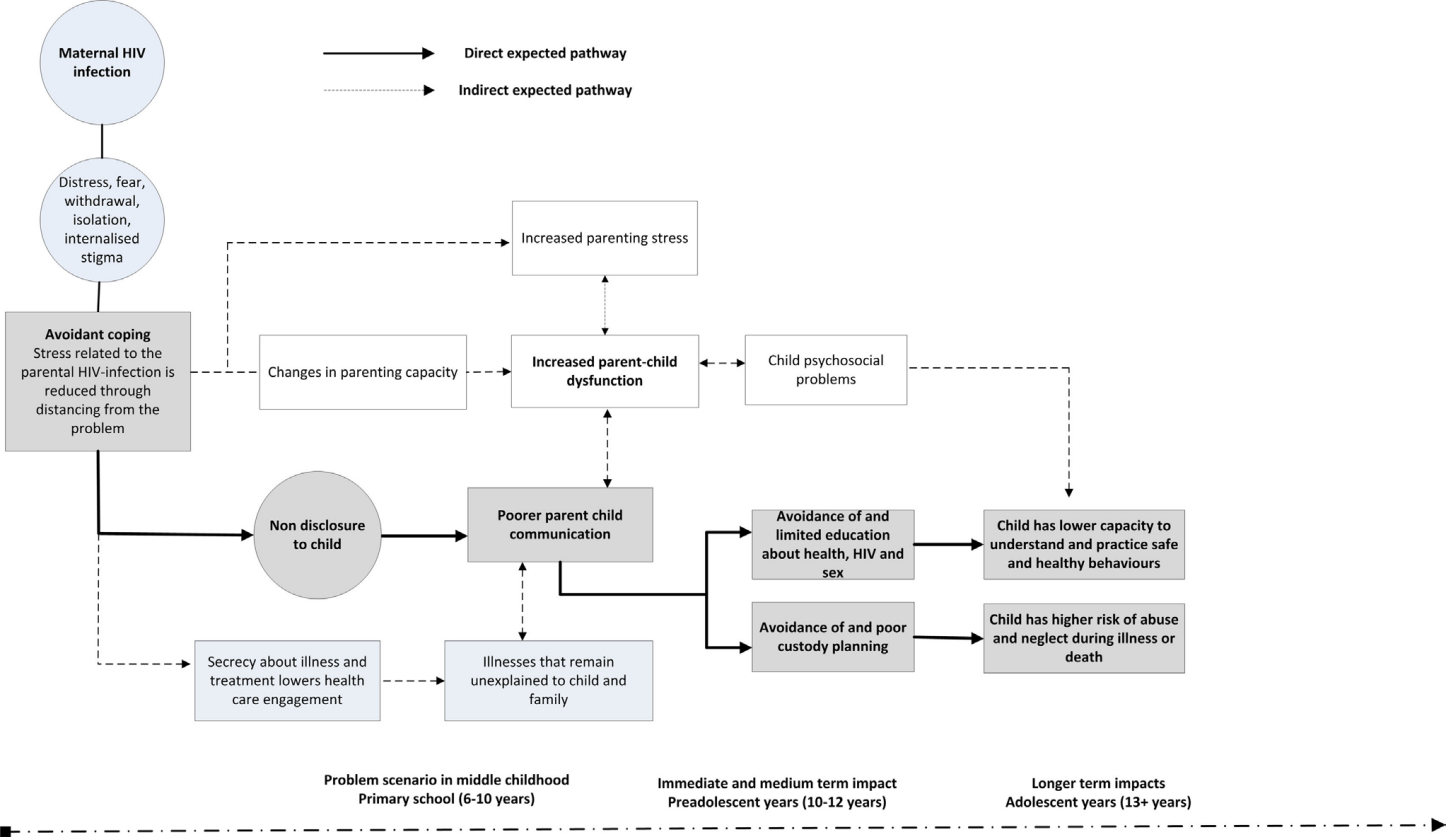
**Pathways to risk for primary school-aged children in the context of parental non-disclosure of HIV status**.

In Figure 1, we outline a developmental framework for the hypothesized risk pathways leading to poorer outcomes among children of HIV-infected mothers. The proposed direct pathway of risk is illustrated in gray boxes; the intervention aims to disrupt this pathway. We hypothesize that maternal HIV-infection, and a lack of openness with the child about it, could lead to psychological stressors for both the parent and child, resulting in negative health outcomes for the child, potentially spanning into the late adolescent years. This conceptual model is described in detail below.

Literature has shown that, following diagnosis, HIV-infected women are known to experience a range of emotions and utilize various coping mechanisms, including what is termed active and avoidance coping strategies (31). Avoidant coping strategies include distraction, denial, escape, distancing, and self-blame, with mothers commonly coping with HIV by distancing themselves from it (32). By not disclosing, which is commonly reported in the HIV literature (18, 33, 34), the mother is practicing a form of avoidant coping, which is often motivated by a desire to safeguard children from psychological distress (26), or concerns about stigma and fear that the child may disclose to others (13, 35).

However, from the broader parental illness literature (36, 37), it is established that even if children are not explicitly informed, they are often aware from their parents’ mood or behavior that something is wrong, or that their parents have concerns which they are not conveying (36, 38–40). Children may blame themselves, internalize their emotions, or exhibit behavioral difficulties (36). This, in turn, increases pressure on the parenting role (41), at a time when HIV-illness and other disease-related stressors are common. This pressure on the parenting role may cause a breakdown in parent–child communication, which negatively affects the mental health of the mother (41, 42) and child (7, 13, 35).

A lack of parent–child communication could result in a lack of health education (43) and lowered care and custody planning for the child (7, 9, 11, 22, 33, 35). It is reasonable to hypothesize that at least some of the risks conferred to HIV-exposed children may be linked to the broader care and custody environment in which the child finds themselves following their parents’ death (6, 44). HIV-infected parents and their families face multiple stressors, including strained family relationships which could complicate care planning for the child (45). Children may be shifted from temporary homes, separated from siblings, or be left with inadequate or inconsistent care (45–48). These happenings may result in children being more vulnerable to abuse and neglect during these periods of illness or death (7, 49, 50).

Literature has also shown that, in the long term, HIV-exposed children may engage in harmful behaviors impacting their sexual health, including earlier sexual debut and risk of transactional sex (50), particularly where children face multiple cumulative risks (51). A lack of good quality parent–child communication may contribute to this, illustrating that the effects of parental HIV in the childhood years have potential to be long lasting and may even increase the risks of the child becoming infected with HIV themselves (50). Therefore, parental HIV disclosure provides an important opportunity for parents to educate their children about their own health and sexual issues.

We propose that through these risk pathways children enter adolescence ill-equipped to manage the risks placed upon them growing up in an HIV epidemic community.

#### Intervention Model

**Figure 2:**
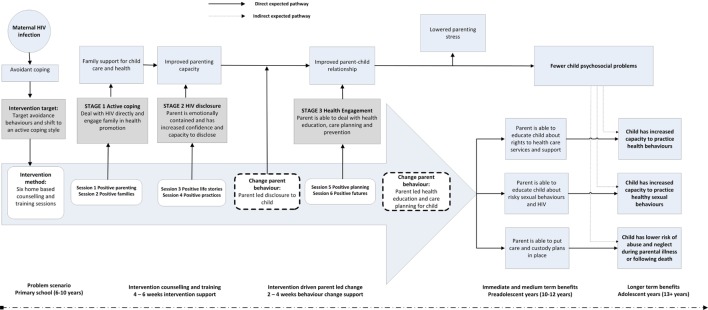
**Amagugu intervention model**.

Figure 2 shows the intervention model, including the stages of the intervention, and how they aim to address and disrupt the pathways of risk outlined in the conceptual model.

The intervention targets parental HIV disclosure as a way to foster active coping, improve parent–child communication and to increase parenting capacity to educate and plan for the child’s future. The intervention directly tries to reduce secrecy and stigma associated with HIV, which may lead to poorer family communication (52), with negative consequences for children (13, 53). Importantly, in contexts where HIV is less prevalent, parents and families may make a choice to keep HIV a secret, and this may have fewer negative consequences for children in particular if it is plausible that they remain unaware, and parents and families remain high functioning (15). However, in an epidemic context, where up to 50% of households have at least one adult living with HIV and taking ART medication (3), it is plausible to assume that parents and families are not able to protect children from the effects of HIV within their family or community (54). Developmental literature from other chronic diseases (37) would, on balance, suggest that developmentally sensitive disclosure of illness is better than non-disclosure, particularly for primary school-aged children. The Amagugu intervention makes use of a variety of psychological approaches, packaged in activities that are accessible and user-friendly for lay professionals. It has five intervention targets that are summarized below, with supporting evidence, and has been specifically developed for high prevalence, low resource, settings.

##### Intervention Target One: Parenting Capacity

Research has suggested that maternal coping strategies are strongly associated with parenting styles and capacities (33). Active coping has been strongly associated with positive parenting, while avoidant coping was linked to poorer quality parenting, and higher externalizing and internalizing behaviors among children (33, 41). Drawing on the parenting literature, we hypothesized that compromised parenting and childcare practices contribute to negative outcomes for HIV-exposed children (38). This process is preceded by HIV stressors impacting negatively on the mental health of mothers, which in turn may negatively affect her parenting capacity (41). Specifically, poor parental mental health is associated with negative child behavior, low perceived parenting capacity, coercive parenting, and low attention to child emotional expression (55, 56). The Amagugu intervention attempts to address avoidant parenting behaviors and increase parenting capacity and skills, which will improve parent–child communication and the quality of the parent–child relationship.

##### Intervention Target Two: HIV Disclosure

Research suggests that disclosure has benefits for mothers in terms of mental health (41, 57, 58), health care behaviors (12), including ART adherence (59), and family relationships (15, 16). While it has been reported that children have an initial emotional reaction following disclosure, in general this is short lived (29), with mothers rarely reporting regrets post-disclosure (13, 18, 60). Furthermore, case–control studies have shown that children who have not been disclosed to show poorer emotional and social functioning (7, 11). Several studies have shown improvements in children’s emotional and social functioning post-disclosure (11, 13), with children reporting feeling better prepared for the future and more involved in decision making. Mothers (14, 61) and children (60, 62) have reported experiencing a closer relationship following disclosure (13, 29); however, there are some studies that do report negative effects (60, 63).

Literature shows that mothers frequently express the desire to disclose to their children themselves (13, 18) but report feeling unsure about how to approach the issue, what is age-appropriate, and often have concerns that disclosure may cause emotional difficulties for their children (28, 64, 65). In most research, mothers emphasized the need for assistance in planning and preparing toward disclosure (2, 13, 18, 34). Ensuring timely, age-appropriate, disclosure of parental HIV status to HIV-uninfected children has been shown to increase the quality of custody and emergency care planning (44, 66).

We hypothesized that intervention support that directly facilitates parental HIV disclosure (whether targeting the mother, father, or caregiver with HIV) has a broader potential to disrupt a variety of parent–child relationship risk pathways. In the Amagugu conceptual framework, HIV disclosure is seen as a key modifiable risk factor that may have both immediate and longer-term benefits.

##### Intervention Target Three: Parent–Child Communication

Research on family resilience suggests that family communication, organization, and belief structures are protective (20, 23, 49). This is particularly important in the context of parental HIV, as one of the negative impacts on children and families relates to repeated illnesses that may lead to hospitalizations and separation of the HIV-infected parent from the child and their family (7, 67). Presently, little is understood about children’s developmental capacity to understand HIV and its health consequences (68). The limited available evidence originates predominantly from high income contexts (28) and suggests that primary school-aged children have few preconceived ideas about the meaning of HIV infection (15), and its potential to cause parental death (69). It is not known how generalizable this is in HIV-endemic settings, but some qualitative evidence suggests that high exposure to illness and death may increase the need for children to develop an understanding of how HIV make affect the human body and cause illness (54, 70). In the absence of an HIV-specific evidence base, it is possible to draw from evidence on the disclosure of other life-threatening illnesses by parents, which highlights that a child’s capacity to cope with parental terminal illness is strongly mediated by developmentally appropriate parental communication about the illness and previous exposures to death (37, 54, 71). Given the high levels of exposure to HIV in South Africa, clear communication about HIV with children is essential.

Parent–child communication about maternal HIV is a highly modifiable risk pathway (72). Thus, we hypothesize that improving parent–child communication, specifically about parental HIV, will likely lead to improvements in the quality of the parent–child relationship, which is known to foster resilience in children (38) and result in lowered parenting stress and fewer child psychological problems.

##### Intervention Target Four: Health Education

Several studies have reported that it is more effective to begin parent–child communication about sex in the pre-teen years or before children reach puberty (73), and before they have developed established patterns of behavior (74–76). Children whose parents talk with them about sexual matters or provide sex education or contraceptive information at home are more likely than others to postpone sexual activity, and earlier education has been shown to improve later sexual health outcomes (77). However, a systematic review on parent–child communication about sex in sub-Saharan Africa (78) reported many barriers to sex education for younger children, in particular a lack of knowledge, skills, and confidence among parents and cultural taboos about discussing sex with children. Parents have a significant opportunity to impact on children’s future sexual risk taking, however, few report doing so. There is strong support in the literature to illustrate that a strong parent–child relationship increases parents’ willingness and ease in talking about sex and that supportive interventions that help parents understand what is developmentally appropriate are beneficial (76, 77). Although Amagugu targets younger children, parental HIV disclosure presents an opportunity for a parent to proactively engage in health education and establish a strong parent–child relationship, which in turn increases the opportunities for parent-led communication about sex as the child matures.

##### Intervention Target Five: Custody Planning

The HIV literature has illustrated that children with custody or guardianship plans in place tend to have better outcomes (79). A custody plan could give children a sense of continuity and predictability after a parent’s death, and some research has shown that proactively engaging with HIV-infected parents makes discussing custody planning more feasible (48, 49). Importantly, care and custody planning should be socially sensitive and culturally appropriate if it is to improve outcomes for children (80). Developing a clear plan for the child’s future care may be a useful adaptive activity that could foster greater family resilience (23) in the context of parental HIV. Research also suggests that increased HIV disclosure is associated with increased custody planning (13). Given these known benefits for children in the longer term, Amagugu targets improving parenting capacity for custody planning, following parental HIV disclosure.

#### Intervention Package

The Amagugu intervention includes an intervention materials package, with session content and activities directly linked to the conceptual framework. These activities draw on evidence from both the family resilience (20, 22) and child development (37, 71) literature.

**Figure 3:**
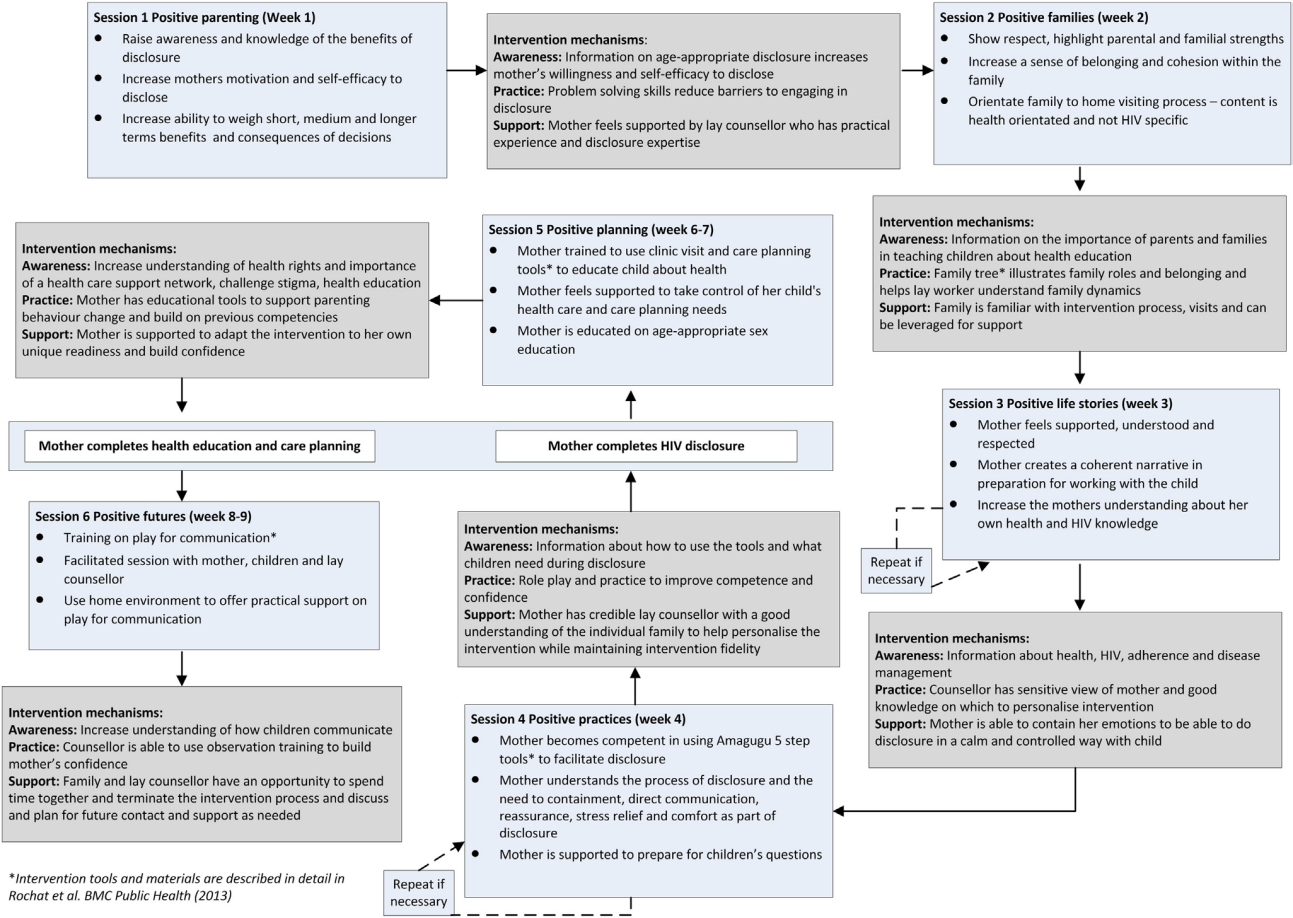
**Intervention overview and mechanisms of change**.

Figure 3 illustrates each session, its content, and the mechanisms by which the intervention aims to bring about change. The content of the intervention is described in detail elsewhere (81). Each intervention session aims to change parenting capacity and behavior through three processes: increasing parental *awareness and knowledge*; increasing parenting *capacity and skill*; and offering *support* in the parenting behavior change process.

During the formative and evaluative work, we developed several key principles that underpinned the approach to be taken in delivering Amagugu:

1. Enhancing parenting capacity is key to change and prevention.
2. Maternal capacity to contain emotions is a precursor to successful disclosure.
3. Parental HIV disclosure of any level is acceptable.
4. Education, care planning, and communication are a pathway to prevention.
5. Flexibility to engage other parental or family figures enhances the intervention.
6. The family is the best context for HIV disclosure.
7. Provision of intervention materials is important to support families.
8. An intervention design that supports a task shifting approach has more potential to be scaled-up.
9. Minimum standards, under which Amagugu is an appropriate and safe intervention, are defined.

##### Parenting Capacity Is the Central Mechanism for Change and Prevention

The intervention targets the parent, with a specific focus on building awareness and knowledge, providing training to increase parenting capacity, and providing support to the parent to undertake disclosure, health education, and care planning with their child. Parenting capacity is fostered through a series of carefully designed preparatory sessions where, after HIV disclosure, education, and planning are led by the parent themselves, and take place independently of intervention counselors, in order to ensure increased skills transference and self-efficacy in parenting capacity.

##### Maternal Capacity to Contain Emotions Is a Precursor to Successful Disclosure

One of the key aspects of the Amagugu intervention is that the mother is supported and enabled to disclose at a time that is suitable for her (without the counselor). It is critical that she is sufficiently prepared and able to contain her own emotions before she would be able to undertake the disclosure with her child. Not only will she need to be able to talk through the diagnosis with her child, but is likely to have to deal with a range of questions, some of which will be difficult, and potentially upsetting, to answer. Thus, the sessions that form Stage 2 (Figure 2) focus on the mother’s feelings about her HIV diagnosis and helping her to come to terms with it and to reach some level of emotional equilibrium. If the mother is still struggling with her feelings at the end of the session, and it appears she may have difficulty disclosing her diagnosis to her child without becoming upset, the session can be repeated.

##### Parental HIV Disclosure of Any Level as the Primary Intervention Target

In Amagugu, parental HIV disclosure is the primary target of the intervention. As the intervention targets children aged from 6 to 10 years, we considered that the intervention would be framed developmentally to ensure scalability and reach. Taking guidance from the literature, Amagugu allows flexibility of parental disclosure level that can be partial (using the word “virus” and not naming HIV) or full (naming “HIV”). Importantly, the level of disclosure is determined by the parent, taking into consideration their child’s developmental needs, their judgment on their child’s level of readiness, and their own level of readiness as a parent.

##### Education, Care Planning, and Communication as a Pathway to Prevention

Parental HIV disclosure is not the only intervention target. We hypothesize that the communication about parental HIV provides an opportunity to increase health education among children and to encourage planning for the child’s future. The intervention, thus, focuses on strengthening parenting capacity to increase health education, care, and custody planning; using activities that support improved communication and quality of the parent–child relationship to confer positive effects beyond the disclosure itself. The intervention, thus, adopts a preventative approach to risks that have been documented to emerge in the context of parental HIV at later developmental stages.

##### Flexibility to Engage Other Parental or Family Figures

The intervention targets mothers specifically, as the vast majority of children are cared for by their mothers in Africa (2). However, the intervention design accommodates involvement of fathers, the mothers’ parents or siblings, the child’s adult siblings, and other family members, alongside the mother (81). In family situations where the mother is not the primary caregiver, the intervention is highly adaptable to alternative primary caregivers.

##### The Family as Context for HIV Disclosure

It is well-established that families can play an important role in resilience in the face of stressful events (20, 82, 83). Families cope by making meaning of the crisis or difficult situation, by developing shared hopes for the future, and by helping children feel connected and problem-solving together (23, 39). Amagugu is developed to take place in the context of the family and includes family activities that foster a sense of belonging and connectedness, and also serve to orientate the family to intervention visits. Family activities are not HIV specific, so they allow mothers to adapt the family activities to suit their family composition and the level of disclosure within the family. Therefore, a key principle of Amagugu is to actively engage family support for the mother as a parent and for her children, regardless of the degree to which family level disclosure has been undertaken.

##### Provision of Intervention Materials

Taking guidance from a successful intervention in the United States (42), and understanding that parents have limited resources in our setting, the intervention provides a set of low-cost materials that are user-friendly and age-appropriate to support disclosure, health education (including sex education), and custody and care planning. The Amagugu intervention materials include storybooks, educational games, and activity cards. Mothers in the pilot study reported finding that the materials increased their confidence to disclose by providing a structure, and being understandable and appropriate for the child (81).

##### Intervention Design which Supports a Task Shifting Approach

In South Africa, and other poorly resourced contexts across Africa, psychosocial interventions at the primary health care level are restricted by critical shortages in health care professionals (84), and the absence of counseling or intervention guidelines (85–87). Task shifting of primary care and prevention functions to community healthcare workers or lay counselors is showing promise in improving health outcomes at reasonable cost (88–90), including examples of cognitive behavioral interventions for postnatal depression (91) and complex treatment regimens, such as ART (92). In the setting where Amagugu was developed, community health care workers and lay counselors are responsible for psychoeducation within HIV treatment programs, including HIV counseling and testing, health promotion, and training of HIV-infected people to take ART. Throughout its development, Amagugu has utilized staff at an equivalent skills level to an HIV counselor to implement this intervention, particularly important for later scale-up given the time constraints on professional health staff within HIV programs (93–95). The package includes a train-the-trainer manual with intervention content, but also offers implementation guidance, minimum standards and community preparation for Amagugu. For larger scale roll-out, the package includes a supervisor’s/implementer’s guide and training video.

##### Minimum Standards under which Amagugu is an Appropriate Intervention

A set of minimum standards were developed to guide when Amagugu would be an appropriate intervention, as opposed to other public health interventions. These include: (i) that the mother or the disclosing parent/caregiver is in reasonable physical health to be able to undertake disclosure in an emotionally contained manner; (ii) that the mother has access to HIV treatment and health care services; if not then these should be prioritized over disclosure support; (iii) that children have access to the parent prior to, and following, disclosure; the Amagugu package offers particular guidance for migrating and working parents; and (iv) that family disclosure and support is feasible and does not introduce risks for the safety of the mother and child.

### Evaluation of Amagugu Intervention

A pre–post-evaluation design study of the Amagugu intervention was conducted (2010–2012) with 281 HIV-infected women and their HIV-uninfected children, aged 6–10 years. This study aimed to evaluate rates of disclosure, and mental health outcomes of mothers and children, following the Amagugu intervention. The study was conducted from the Africa Centre for Population Health in rural KwaZulu-Natal, South Africa. The methodology and results are described in two open access papers in the journal AIDS (25, 26), and a brief summary of the results are given below. Written informed consent was obtained from mothers/caregivers and assent from children, and ethical approval was obtained from the Biomedical Ethics Committee of the University of KwaZulu-Natal (Ref: BF 144/010).

Amagugu was found to be effective in supporting maternal disclosure. Prior to the intervention, the majority of mothers 234/281 (83%) had not disclosed to any of their children under the age of 18 years, highlighting the need for disclosure support in this age group. Among the 47 mothers who had made a previous disclosure to a child, 21 (45%) had disclosed to an older child aged 10–18 years, while 26 (9%) had disclosed to a younger child aged 6–10 years. Encouragingly, post-intervention, all mothers undertook some level of disclosure, with 61% of mothers fully disclosing their HIV status and 39% undertaking partial disclosure (using the word “virus” as opposed to “HIV”).

In the evaluation, we also demonstrated improvements in maternal and child mental health, with the intervention significantly reducing parenting stress, and children showing less emotional and behavioral difficulties post-intervention (25, 26).

The mean age of children in the sample was 7 years (range from 5 to 10 years); we found age not to be significantly associated with level of disclosure. The majority of children’s reactions to disclosure were reported by the mother to be “calm,” regardless of whether disclosure was partial or full.

An examination of data on the questions that children asked following disclosure (while limited to maternal report) revealed that children have the capacity to understand and engage with the concept of HIV as a disease from a young age. Post-disclosure, children asked questions about the nature of HIV, how it was transmitted, how treatment worked, and how they could prevent themselves from getting infected (25). The evaluation results support the hypothesis that HIV disclosure can be a starting point for health and sex education with younger pre-adolescent children. While sex education was not directly addressed in Amagugu, at baseline 126 (45%), mothers reported having discussed the risks of sexual abuse with the child; post-intervention, this increased to 247 (88%). Sex education tools, including a storybook, have been incorporated in the post-evaluation revised materials package.

Post-intervention, mothers were asked what aspects of the intervention they found most enjoyable, and the results are shown in Table 1. These responses revealed two overarching categories: reflecting enjoyment of the feelings and emotions that the intervention brought about (127/281; 45.2%), and satisfaction with the materials and activities used in the intervention (150/281; 53.4%). In the feelings and emotions category, the most common response was the enjoyment of experiencing the child’s positive reaction to disclosure (67/127; 52.8%), and in the materials and activities category, mothers most frequently expressed enjoying the child-friendly games, storybook, and storytelling activities (64/150, 42.7%). Very little research in the field has explored participant satisfaction with disclosure interventions. As maternal confidence has been shown to be a determinate of disclosure (42), understanding maternal satisfaction – specifically which activities she enjoyed and whether she experienced regrets – is important as they may affect maternal confidence and intervention success. Evaluating maternal satisfaction may also allow greater understanding of the maternal experience and the wider application of the intervention. When asked about their regrets, 274 mothers stated they had none, five stated regretting the child’s reaction to disclosure, one acknowledged initial regret as the child appeared alarmed after the disclosure (but shortly afterwards the child appeared to be fine), and another stated she regretted disclosing “partially” instead of “fully” to her child. When asked what they found least enjoyable about the intervention, 30 (11%) cited the child’s initial reaction, 10 (4%) cited the having to state the disclosure out-loud to the child, 42 (15%) cited some aspect of the using the disclosure materials, while the remaining 199 (71%) cited there was nothing they did not enjoy, suggesting that the intervention is highly acceptable in this population.

**Table 1:** **What mothers enjoyed most about the Amagugu Intervention**.

Categories of most enjoyable aspects	
**Feelings and emotions, *N* = 127/281 (45.2%)**	
Experiencing the child’s positive reaction to disclosure	67 (52.8%)
Pride in the opportunity to be able to educate and support their child	37 (29.1%)
Feeling relief and a sense of acceptance and care from their child	23 (18.1%)
**Materials and activities, *N* = 150/281 (53.4%)**	
Child-friendly games, storybook and storytelling activities	64 (42.7%)
HIV body map educational tool and health promotion playing cards	40 (26.7%)
The combination of materials and how they fitted together in a package	46 (30.7%)
**Missing, *N* = 4/281 (1.4%)**	

When asked if they had involved family members at different parts of the intervention; 17 and 16% of mothers reported having included family members in preparing to disclose and during the disclosure process, respectively. However, encouragingly, 42% of mothers reported that they had involved family post-disclosure, suggesting that the intervention makes mothers feel more confident to involve others and discuss their HIV status. The intervention indicates potential for the involvement of men, as, of the family members included post-disclosure, almost a third were men, most commonly a brother or a boyfriend. The sample characteristics and relationships of the mothers are described in detail elsewhere (25). Adding to evidence of increased confidence, around 90% of the mothers reported that they felt they could help other mothers in their community to disclose to their children.

## Discussion

Through the results of the Amagugu intervention, we provide evidence to support our conceptual and intervention model. We have demonstrated that children in this context have the capacity to understand and engage with the concept of HIV as a disease from a young age. Some HIV disclosure-related qualitative research (96) has found that while most mothers only disclose to children aged 7 or 8 years, many report that their children had been aware of illness-related information for at least 3 years prior to the disclosure. In this high HIV prevalent context, it seems likely that more children would have prior experience of illness and death, which may account for children’s understanding of HIV and death. This finding is in line with qualitative research in South Africa, which showed that pre-school children have a naïve understanding of human biology and disease, and in a context of high exposure to death, children are likely to assimilate experience and understanding of both external and internal causes of death at a young age (54). This provides evidence supporting the appropriateness of the HIV-related materials in the Amagugu intervention for this primary school-aged group of children.

The evaluation results support the hypothesis that HIV disclosure can be a starting point for health and sex education with younger pre-adolescent children. This is encouraging as existing literature from high income settings shows that parent–child communication about sex can influence later sexual outcomes of the child (73, 74, 97). A recent systematic review (98), examining the impact of behavioral interventions involving parent–child communications about sex in children who are disproportionately affected by HIV in the United States, showed that 13 out of 15 studies showed at least one significantly improved sexual health outcome compared with controls. Likewise a systematic review of the impact of sex education and HIV education interventions in schools in developing countries (97) showed that 16 of the 22 interventions significantly delayed sexual debut, reduced the frequency of sex, decreased the number of sexual partners, and increased the use of condoms or contraceptives.

Parental disclosure is by no means made easy through Amagugu, and it remains a challenging and emotional task for parents, children, and family, but this intervention illustrates that with appropriately targeted support mothers can undertake disclosure and encourage other healthy behavior changes in their children. Results support the flexible nature of the intervention to include alternative caregivers and family members, with almost half the mothers reporting involving other members of their family post-disclosure. Furthermore, the overwhelming majority of mothers reported feeling confident to help other mothers in their community to disclose, illustrating the potential for the intervention to show sustainable benefits, not only for the family and child but also for the wider community.

Limitations of this research include lack of costing of the intervention package, no control group, and parent-reported data on children’s mental health. Furthermore, as a minimum standard we only included mothers who had access to HIV treatment services, which may have limited our sample. The intervention requires further testing with other caregivers (for example HIV-infected fathers) and in other settings.

Amagugu has shown preliminary success in a large-scale evaluation, but these results must be interpreted with caution, given the absence of a control group. In 2013, with funding from the National Institutes of Health (RO1HD074267-01), Amagugu was tested in a randomized control trial (NCT01922882). This trial was completed with follow up to 9 months post-disclosure in December 2015 and results will be published in 2016.

## Author Contributions

TR contributed to drafting the manuscript and to the design of the conceptual framework and intervention design, critically revised the manuscript, and contributed to analysis and interpretation of evaluation data. JM contributed to drafting and critically revising the manuscript. AS contributed to revising the manuscript and to the design of the conceptual framework and intervention design. NM contributed to design and data collection and to drafting and critically revising the manuscript. RB contributed to the design of the conceptual framework and intervention design, critically revised the manuscript, and contributed to analysis and interpretation of evaluation data.

## Conflict of Interest Statement

The authors declare that the research was conducted in the absence of any commercial or financial relationships that could be construed as a potential conflict of interest.
